# Elements of General and Pathological Anatomy, Presenting a View of the Present State of Knowledge in These Branches of Science

**Published:** 1851-11

**Authors:** 


Art. VI.—*Elements of General and Pathological Anatomy, presenting a view of the present state of knowledge in these branches of Science.* By David Craigie, M.D., F.R.S.E., Fellow of the Royal College of Physicians, Edinburgh, and Honorary Consulting Physician to the Royal Infirmary. Second edition, enlarged, revised, and improved. Philadelphia: Lindsay & Blakiston. 1851. 8vo. pp. 1072.

This elaborate work differs in some important particulars from most of the systems of pathological anatomy. The basis of arrangement chosen by the author are the distinctions of the component tissues of the animal body, as derived from the similitude and difference of their anatomical characters. He gives, first, a division of the tissues; then describes the fluids and their morbid states; and then, successively, the various tissues, cellular, adipose, venous, and so on, in connection with the diseases incident to them. It is a work of great labor and great science, from which men well versed in the profession as well as students of medicine may derive large stores of knowledge. As stated in the title-page, the author has attempted to exhibit "a view of the present state of knowledge in the branches of science" to which it relates; and we know not where we could refer for a more full and complete exhibition of the sciences of general and pathological anatomy than is afforded by his work. The reader who has the courage and industry to sit down to the study of so ponderous a volume may be sure of quitting it with his mind stored with the best fruits of research in these interesting branches of medicine. In this light we cordially recommend it, and shall be gratified to learn that it has been extensively read. It affords a compend of knowledge on these subjects quite as enlarged as the practitioner could desire, communicated in a most perspicuous and methodical manner. The mechanical execution of the volume is excellent, and if it would have been on some accounts more valuable for being illustrated, it is cheaper for being without illustrations. Y.

